# Experiences of Chinese patients with Crohn’s disease in the self-administration of nasogastric feeding: A descriptive qualitative study

**DOI:** 10.1371/journal.pone.0201421

**Published:** 2018-07-30

**Authors:** Qian Cai, Fang Li, Yunxian Zhou

**Affiliations:** School of Nursing, Zhejiang Chinese Medical University, Hangzhou, China; University Hospital Llandough, UNITED KINGDOM

## Abstract

**Background:**

Despite the increasing number of Crohn's disease patients self-administering nasogastric feeding as enteral nutrition support therapy, no studies have reported the experiences of self-administering nasogastric feeding from the perspective of these patients.

**Objectives:**

To explore the initial trigger factors for the self-administration of nasogastric feeding by Crohn’s disease patients and to understand the experiences of self-administration of nasogastric feeding, its effects on various aspects of life and work, and relevant challenges and coping mechanisms encountered during this therapy in order to improve the understanding of this group of patients among medical staff and the public.

**Design:**

This study adopted a descriptive qualitative method. Crohn's disease patients from several tertiary hospitals in Hangzhou, Zhejiang, were recruited to participate through purposive sampling combined with maximum variation and the snowballing technique. Data were collected using semi-structured interviews and analyzed using the conventional content analysis method.

**Results:**

A total of 11 Crohn's disease patients were interviewed. Four themes and eight subthemes emerged from the analysis: rejecting the self-administration of nasogastric feeding (being afraid of inserting the nasogastric tube, having concerns about nasogastric feeding), accepting the reality of nasogastric feeding (health being the most important, followed by having sources of support), nasogastric feeding as a double-edged sword (the disturbances and efficacies of nasogastric feeding), and nasogastric feeding as a part of life (becoming accustomed to tube insertion and taking nasogastric feeding for granted).

**Conclusions:**

Apart from suffering from physical discomfort, diet and body image disturbances, and inconveniences in daily life, Crohn's disease patients who self-administered nasogastric feeding faced many psychological challenges. Many of these patients eventually adjusted to a life with nasogastric feeding, but not everyone achieved this state. Therefore, health care providers, including physicians and nurses, and the general public should collaborate to help these patients adapt to their “new lives” as soon as possible.

## 1. Introduction

As an inflammatory bowel disease (IBD), Crohn’s disease (CD) is a chronic relapsing gastrointestinal condition that has an unknown etiology and is currently incurable [1–3]. Since CD can affect all parts of the digestive tract, patients are prone to digestive and nutrition-absorption disorders [3]. The malnutrition rate in hospitalized surgical patients with CD is as high as 86.7%; moreover, malnutrition can reflect the severity of the disease [4] and influence quality of life and long-term survival rates [5, 6]. Enteral nutrition (EN) is not only effective in improving the nutrition status of CD patients, but it also has a role in promoting mucosal repair and improving the natural course of the disease [7–9]. Guidelines and expert consensuses from many countries, including China, have indicated that nutrition support should be part of the holistic treatment of CD patients [7, 10,11].

As nutrition agents and nasogastric tubes have improved, the application of nasogastric feeding has become increasingly widespread [12]. Currently, under the supervision of health care providers, many CD patients have adopted home EN support [13]. After mastering the corresponding skills, patients can insert the nasogastric tubes by themselves at night or in their free time for feeding. Therefore, CD patients are capable of meeting their nutritional needs and managing the feeding time based on their own circumstances.

Studies on nasogastric feeding of CD patients have mainly focused on clinical efficacies or illness experiences. For example, in the study by Robinson, nasogastric feeding was helpful in improving the body symptoms of IBD patients [14]. According to Aiges et al., the self-administration of nasogastric feeding at night might guarantee a normal diet intake and activities during the day, thus minimizing the impact of the disease [15]. However, nasogastric feeding has also been reported to cause physical discomfort, reduce self-image, affect the quality of life and result in tube management issues [16–19]. Basson found that the feeding dose should be adjusted based on the patient’s condition; otherwise, it can cause symptoms such as hunger or diarrhea [20]. In addition, in the study by Palant et al., patients with IBD who were unable to consume food normally experienced substantial psychological stress [21]. These negative experiences can be minimized if patients receive adequate understanding and support from others [22]. Meanwhile, CD patients initially thought that the disease controlled every aspect of their lives because of the dietary restrictions, but they gradually develop a positive view toward long-term nutrition support and were proactive in enacting effective coping mechanisms [23].

To date, few studies have examined the experience of patients who administer self-inserted nasogastric feeding. Therefore, this study aimed to explore the trigger factors for the initiation of self-administering nasogastric feeding, the problems and difficulties encountered and their relevant countermeasures during nasogastric feeding, and how nasogastric feeding influences the daily life of CD patients. We anticipate that the findings from this study will enhance the understanding of this group of patients for both health care providers and the general public. It can provide a basis to develop clinical interventions and management plans for this population and to help them acquire more understanding and support from society.

## 2. Methods

### 2.1. Study design

A descriptive qualitative design was adopted [24]. In this approach, researchers avoid using preconceived categories and immerse themselves in the data to enable new insights to emerge that provide a richer understanding of the phenomenon [24].

### 2.2. Participants

A purposive sampling approach, combined with maximum variation (gender, age, occupation and duration of nasogastric feeding) and the snowballing technique, were used to select CD patients from several tertiary hospitals in Hangzhou, Zhejiang, from February 2016 to December 2016. Eligible participants displayed the following characteristics: (1) diagnosed with CD based on the Chinese criteria released in 2012 [2]; (2) aged between 18 to 60 years old; (3) self-administered nasogastric feeding at least once a day and for at least 3 months; and (4) volunteered participate in the study with no communication barriers. Participants were excluded if they had other chronic diseases (such as hypertension, diabetes, and coronary heart disease), cancer, or were pregnant at the time of the study.

### 2.3. Data collection

Data were collected using semi-structured interviews that were generally conducted face-to-face. Initially, an interview guide was developed based on a literature review, which focused on the following five questions: (1) How did you feel when you were informed that you needed to receive nasogastric feeding therapy? (2) What were the trigger factors for you to start self-administered nasogastric feeding? (3) How did you feel when you tried nasogastric feeding for the first time? (4) What were the memorable events that you encountered during your self-administered nasogastric feeding? (5) What effect has self-administered nasogastric feeding had on your life and work? Interviews were conducted in private and quiet places chosen by the participants and were recorded. At the beginning of the interviews, the researcher introduced herself and had casual chats with the participants to create a relaxing atmosphere. Follow-up questions were used to encourage the elaboration of responses when necessary. Field notes recorded details of the observations, interactions, environment and body language of the participants. The researcher remained neutral and avoided making any judgment on the interview contents. The average length of the interviews was approximately 45 minutes. If necessary, telephone calls were made to clarify the responses of some participants.

### 2.4. Data analysis

After one interview was completed, it was transcribed verbatim. The data were analyzed concurrently with the data collection using the conventional content analysis method [24, 25]. The first step involved reading the entire transcript of the interview several times to obtain an overall understanding of the participant’s experiences. Then, any narrative data that related to the CD patients’ experiences of self-administering nasogastric feeding were hand-coded line-by-line. Next, the generated codes and concepts were grouped into subthemes and themes based on comparisons regarding their similarities and differences. Finally, a definition for each theme and subtheme was created, and supportive quotes from the data were selected. The data collection and data analysis procedures were conducted repeatedly until reaching saturation, with no further themes or subthemes emerging [26].

### 2.5. Rigor

In this study, rigor was maintained using the two methods. First, credibility was established through peer debriefing, in which the researchers consulted with one another to address any ambiguities or disagreement on methodological issues or data analysis. Second, the themes and subthemes extracted from the collected data were repeated to the participants to further confirm whether they had the same feeling or experience and record any possible supplemental information. Three participants were involved in the member checking process.

### 2.6. Ethical considerations

Ethical approval for this study was obtained from the ethics committee of Zhejiang Chinese Medical University, and written informed consent was obtained from each participant. All participants were informed about the purposes of this study. They were also informed that they had the right of refusal and that this right could be exercised at any time without having any negative impacts on the services delivered to them. We also guaranteed the confidentiality of their personal information.

## 3. Results

A total of 11 CD patients who self-administered nasogastric feeding were recruited for this study. Four patients were male, and seven were female, ranging in age from 26 to 58 years. Detailed demographic and clinical information is shown in Table 1. The analysis yielded four themes and eight subthemes (Table 2).

**Table 1:** Demographic and clinical characteristics of the participants.

Participant	Gender	Age (years)	Occupation	Marital status	Education level	Course of disease (years)	Duration of nasogastric feeding (months)
1	Female	43	Unemployed	Married	Senior high school	7	3
2	Female	38	Self-employed	Married	Senior high school	3	4
3	Female	30	Freelancer	Married	Bachelor’s degree	2	3
4	Female	58	Teacher	Married	Bachelor’s degree	10	12
5	Male	28	Accountant	Single	Bachelor’s degree	2	4
6	Male	26	Engineer	Single	Master’s degree	1	9
7	Male	38	Accountant	Married	Bachelor’s degree	10	4
8	Male	40	Staff	Married	Associate’s degree	2	10
9	Female	27	Self-employed	Married	Bachelor’s degree	5	36
10	Female	39	Worker	Married	Junior high school	9	12
11	Female	39	Civil servant	Married	Master’s degree	3	18

**Table 2:** Themes and subthemes of the study.

Themes	Subthemes
Rejecting the self-administration of nasogastric feeding	Being afraid of inserting the nasogastric tube
	Having concerns about nasogastric feeding
Accepting the reality of nasogastric feeding	Health being the most important
	Having sources of support
Nasogastric feeding as a double-edged sword	The disturbances in nasogastric feeding
	The efficacies of nasogastric feeding
Nasogastric feeding as a part of life	Becoming accustomed to tube insertion
	Taking nasogastric feeding for granted

### 3.1. Rejecting the self-administration of nasogastric feeding

The rejection of the self-administration of nasogastric feeding referred to the experience that CD patients had when they were initially informed that they might need to adopt self-administering nasogastric feeding as nutrition support therapy. The patients rejected this idea because they were afraid of inserting the nasogastric tube, and they had concerns about nasogastric feeding.

#### 3.1.1. Being afraid of inserting the nasogastric tube

Patients who self-administer nasogastric feeding must insert a nasogastric tube of a certain length into the stomach through the nasal cavity, which is a horrible thing to imagine for those who have never tried it before or tried it only for the first time.

*I was afraid of self-inserting the nasogastric tube*. *Just imagine that a tube is being inserted from your nose to your stomach and you need to perform the whole procedure; this process (is quite horrible)*.

(P8)

The fear felt by CD patients was mainly caused by a lack of understanding of the process for self-insertion of the nasogastric tube. Some patients had absolutely no idea of how nasogastric feeding therapy worked, whereas others had no personal experience with it.

*When she (the doctor) told me to consider nasogastric feeding therapy*, *I was very astonished and responded*, *“What is nasogastric feeding for?” I had no idea about that …no idea at all*. *I was frightened to death at that time when I heard that a tube was going to be inserted into my body*.

(P2)

The fear was aggravated if the CD patients had previous bad experiences with inserting tubes, particularly painful experiences.

*Prior to nasogastric feeding*, *I was hospitalized*. *A tube (gastrointestinal decompression tube) was inserted into my stomach to suction out my stomach fluid*, *which was very painful*, *and it caused me to experience a psychological disorder*. *Because of that fear*, *the insertion of any tube would make me uncomfortable…So*, *I had a psychological disorder related to nasal tube insertion*.

(P4)

Some CD patients already had an imaginative fear of tube insertion; they were even more worried if they heard that another patient made a mistake regarding the insertion position.

*I’ve heard that a tube was once inserted into the lung by someone…Particularly when you were inserting by yourself without health care providers nearby*. *I was worried because I had no idea what reactions I would have if the tube was wrongly inserted or whatever*.

(P9)

The fear of self-insertion of the tube was intensified if the CD patients had witnessed that other patients succeeded in tube insertion only after several trials and experienced physical discomfort during the insertion.

*That patient only successfully inserted the tube after a long trial*, *which scared me to death…He had nausea while inserting the tube*. *I was nervous when seeing this because he failed to insert the tube correctly several times*.

(P2)

#### 3.1.2. Having concerns about nasogastric feeding

In China, the self-administration of nasogastric feeding is only used by a particular group of CD patients, and many patients are not familiar with the procedure. Furthermore, because nasogastric feeding would change their original lifestyle, CD patients had many concerns, including concerns about disease severity, body image, daily diet and daily activities. The extent of the concerns was associated with the degree to which the patient rejected nasogastric feeding.

Concerns about disease severity referred to CD patients’ belief that the nasogastric feeding therapy would be necessary only if their conditions were severe. They did not consider their disease condition to be that severe; however, doctors advised them to try nasogastric feeding. This contradiction resulted in a psychological rejection of nasogastric feeding.

*At the beginning*, *I rejected this therapy a lot…and thought that the patient who inserted a nasogastric tube was the one in serious condition (laughing)*. *They were old patients or those who needed to be rescued…Rejection was the result of unwillingness*, *because I was not willing to accept the fact that I had reached such a severe level*.

(P3)

Concerns about body image were related to the distinct image of CD patients with a feeding tube compared to normal people. This difference generated a certain level of psychological stress in patients, and the degree of the stress was associated with the nature of the patient’s occupation.

*I was engaged in the service industry…Every day*, *I was faced with customers from all over the country*, *so that I must pay enough attention to my image*.

(P2)

Concerns about daily diet when engaged in nasogastric feeding prevented CD patients from eating normally, which caused them to feel that “I am not the same as others” and brought psychological stress.

*For instance*, *you were administering nasogastric feeding by yourself while others were eating/having meals*. *It seemed that you were separated from others*.

(P7)

Another concern was restricted activities. Since CD patients who self-administered nasogastric feeding were required to tube feed a nutrition solution, it was inconvenient for them to go out, and the space in which they could perform activities was restricted accordingly.

*You needed to hang a bottle like an intravenous drip when adopting nasogastric feeding*. *Naturally*, *the activity space was restricted…lying on the bed all day*, *with a bottle hung up*.

(P7)

Other concerns were caused by an insufficient understanding of nasogastric feeding. Some patients incorrectly believed that frequent hospital visits were required after adopting nasogastric feeding therapy.

*(I used to think that) I had to go to see a doctor every day for the tube insertion*. *What a trouble it could be*! *It was hard for me to deal with*.

(P3)

### 3.2. Accepting the reality of nasogastric feeding

Some patients accepted the reality of nasogastric feeding after they persuaded and/or encouraged themselves to accept the reality of nasogastric feeding after receiving some preliminary information about it and going through a period of inner struggle. The awareness of health as the most important factor and having sources of support promoted acceptance.

#### 3.2.1. Health being the most important

CD patients preferred to accept nasogastric feeding for recovery and disease control. When they were aware of the severity of their illness, they accepted nasogastric feeding therapy in a relatively calm manner to prevent potential exacerbations.

*It was not until the third time I visited the XX hospital that I decided to accept nasogastric feeding therapy*. *By that time*, *my condition was relatively severe*, *and I already had some intestinal obstruction; thus*, *I finally decided to adopt nasogastric feeding*.

(P11)

CD patients were more proactive in attempting the self-administration of nasogastric feeding when they had a certain understanding of the therapy and had a high expectation of its effect.

*There is an old saying in China that gastrointestinal disease relies on bowel resting*. *Nasogastric feeding*, *to some extent*, *sounds like a bowel resting method*. *Well*, *at that time*, *I hoped that I was the lucky one and this therapy could help me to recover from the illness*.

(P5)

When CD patients were informed that nasogastric feeding therapy had substantially similar effects as hormones, immunosuppressive agents, etc., but the latter drugs could cause some side effects that nasogastric feeding did not, nasogastric feeding seemed to be a better option.

*The nutrition support therapy did work*, *and it was capable of remitting the intestinal flares without side effects compared to some medicines*, *which might cause side effects such as vomiting or the like*. *Why I did not try this safe and side-effect free therapy?*

(P3)

When struggling to decide between nasogastric feeding therapy and surgery, CD patients typically preferred nasogastric feeding after considering the high risk of surgery and high recurrence rate.

*Considering the risks of surgery*, *one risk was the operation itself*, *and another was the recurrence rate after surgery*. *The disease could not be cured by an operation anyway and the recurrence rate was very high*, *that is*, *the probability of needing a second surgery was relatively high*. *Therefore*, *I would rather choose nasogastric feeding therapy instead of surgery*.

(P8)

Compared with oral EN support, CD patients indicated that nasogastric feeding had better effects because it did not require the patient to taste the nutrition solution and therefore it was easier to tolerate and ingest an adequate amount. In addition, CD patients preferred nasogastric feeding therapy to parenteral nutrition support because the former was relatively cheap.

#### 3.2.2. Having sources of support

Having sources of support refers to the inner strength and external support that helped CD patients accept the reality of the self-administration of nasogastric feeding. Three main aspects of support were identified: support from health care providers, support from peers, and personal strength.

The support from health care providers referred to the knowledge and skills of health care providers who provided guidance related to nasogastric feeding that was targeted to CD patients.

*Initially*, *I had no idea about the nasogastric feeding tubes*. *The nurse was quite kind*, *and she taught me carefully…also I watched a teaching video on nasogastric feeding*.

(P2)

Professional guidance provided by health care providers can ease the fears and worries related to tube insertion, and a good attitude can also reduce the potential rejection of nasogastric feeding therapy by CD patients.

*The nurse spoke gently with a good attitude and smiled…as a result*, *the psychological rejection of the patient would be eased to a certain degree*.

(P4)

Peer support referred to support from other CD patients who self-administered nasogastric feeding. The psychological distance from others was minimized when CD patients knew that they were not the only one adopting nasogastric feeding, which enhanced their acceptance.

*When I saw someone similar to my age who was using nasogastric feeding*, *with nasogastric feeding tube on*, *the psychological difference was eliminated…And I realized that I was not a kind of alien anymore*.

(P3)

When they witnessed other patients successfully insert the tubes, CD patients’ fears of tube insertion were reduced, and they were more willing to try to self-insert the nasogastric tube.

*However*, *I saw other patients self-administering nasogastric feeding without as much pain as I had imagined*. *I saw them inserting the tube in and pulling it out from time to time (laughing)…(I felt) it was not that hard to accept (nasogastric feeding)*.

(P1)

Hearing that peer patients’ conditions improved after self-administering nasogastric feeding inspired other CD patients to actively try it.

*I have heard that the nasogastric feeding treatment was effective and safe many times*. *Thus*, *I thought I might have to overcome the barrier (fear of tube insertion) and moved forward*.

(P4)

Inner strength refers to the power retrieved after succeeding in insertion, which enhanced the confidence in administering the nasogastric feeding therapy.

*My first time at inserting the tube was lucky*, *and I succeeded on the first try*. *Therefore*, *it was easy for me to accept the nasogastric feeding treatment…and I was confident inside and felt it was not that hard*.

(P2)

### 3.3. Nasogastric feeding as a double-edged sword

Nasogastric feeding is a double-edged sword, which means that CD patients experience two sides of the therapy: the disturbances and the efficacies. Accordingly, if the disturbance of nasogastric feeding dominates, adherence is affected. Moreover, if the patients consider the efficacies to be greater than the disturbances, they are more likely to adhere to the self-administration of nasogastric feeding.

#### 3.3.1. The disturbances in nasogastric feeding

The disturbances encountered during self-administration of nasogastric feeding were mainly caused by changes in normal living conditions and the difficulty in coping with those changes. The disturbances included physical discomfort, an inability to consume food normally, an abnormal self-image, and increased inconveniences in daily life.

Nasogastric feeding caused a certain degree of physical discomfort, which usually occurred in the early stage, and it mainly included nasopharyngeal discomfort and diarrhea.

*I had loose bowels*, *and diarrheas…all the time*, *sometimes two or three times a week*, *or sometimes four or five times a week*.

(P6)

The physical discomfort was exacerbated if the patient had a cold or suffered from rhinitis.

*I have allergic rhinitis and my nasal cavity was quite uncomfortable*. *On the first and second days of tube insertion*, *the interior of my nose was not very comfortable with a runny nose*. *Moreover*, *the stuff (tube) kept stimulating the nose and the runny nose due to allergic rhinitis (got more severe)*.

(P4)

Due to the nasogastric feeding, CD patients were unable to have meals together with their families and friends, which caused a certain degree of psychological stress. The patients felt distressed about being unable to meet their own appetite, particularly when they saw others having meals.

*In our ward*, *there was someone …*, *anyway*, *a patient in the same room*. *Her husband was eating instant noodles in front of us who were unable to eat*. *When she was seeing this*, *she asked him to go outside to eat so that we would not see it*. *It was quite annoying for us to see someone eating in front of us*.

(P10)

Moreover, the annoyance was aggravated if their colleagues and family members did not understand them.

*My colleagues kept asking me*, *“Hey*, *do you want to eat something? I found that you haven’t eaten for a quite long time*, *are you hungry?” They often asked me such questions*, *which were quite annoying*, *yet I could not get angry with them and I could only explain to them by saying “I am not hungry*, *I am ok” repeatedly…You know that they would never be able to understand you*.

(P5)

The presence of the nasogastric feeding tube in the nose altered the physical appearance of CD patients compared to a healthy person. This abnormal appearance caused a certain degree of psychological stress in the patients.

*When the tube was inserted*, *I felt quite strange since a small portion of the tube was exposed outside in such a manner that I felt myself a bit like an elephant*, *with a long nose extended outwardly*. *I was a bit (at a loss)*, *having no idea of how to deal with that*.

(P11)

In addition, other people might exaggerate the severity of the illness because of the appearance of the nasogastric tube and think that the CD patient had an “incurable disease”, “a very terrible disease” or “a terminal illness”, which increased the psychological burden on the patient.

*When I met other people*, *they (friends nearby) thought I was very strange and sometimes would even tell me that they considered I was close to dying (with such tube) (laughing)*. *To be honest*, *though I did know that there was nothing serious with the tube*, *others would think I was in a critical situation*.

(P5)

The need to self-administer nasogastric feeding caused inconveniences in the daily life of CD patients. For instance, patients were required to carry a nasogastric feeding pump (or gravity tube) and bottles of nutrient solution with them when going out, which was inconvenient.

*I must carry such stuff around all the time*, *which is quite troublesome*. *No matter where I go*, *I must take such stuff here and there*, *which is very inconvenient*.

(P5)

In addition, nasogastric feeding at night would affect sleeping quality or even sexual life of CD patients.

*After one day of tiring work*, *I returned home*, *it would be troublesome for me to do this*, *and especially when I had to get up at midnight (to change the nutrition solution)*. *It ruined my resting schedule because I was worried about when to change another bottle*. *With these problems*, *the sexual life was certainly influenced*, *right?*

(P2)

#### 3.3.2. The efficacies of nasogastric feeding

After a period of nasogastric feeding treatment, CD patients felt that their symptoms and their quality of life had improved. The efficacies include two aspects: subjective symptom improvement and better examination results.

Subjective symptom improvement refers to weight gain, better skin condition, and improvements in gastrointestinal discomfort, such as indigestion, constipation, diarrhea, and abdominal pain.

*I was 15 kilograms heavier than before*. *Previously*, *three bottles a day*, *now I changed to two bottles (laughing)*. *If I keep on gaining weight like this*, *I might need to lose weight*, *so I reduced the dosage of tube feeding*.

(P8)

*After adopting nasogastric feeding for a certain time*, *my skin became very clean and it looked like that I have changed to be another person*.

(P1)

*It was hard for me to digest the food I ate*, *usually just poo what I eat*. *After nasogastric feeding*, *this gastrointestinal discomfort disappeared …When I nasogastric fed and I didn’t eat anything else*, *I would feel very comfortable*.

(P7)

After a period of nasogastric feeding treatment, the laboratory and imaging results of the CD patients also improved. Laboratory indicators, such as hemoglobin and fecal calprotectin, normalized. Better imaging results were mainly reflected in the colonoscopy and small intestine computed tomography results.

*My hemoglobin test result was unprecedentedly good*. *Previous results indicated that I had anemia*, *eight grams*, *nine grams*, *ten grams or the like … now the test result indicated 13 grams of hemoglobin*.

(P3)

*I had a lot of ulcers when I had colonoscopy examinations before*. *However*, *the ulcers disappeared after using the nasogastric feeding treatment*.

(P11)

Symptom improvement and better laboratory results allowed the CD patients to return to a normal life and re-achieve their own values.

*Now I am almost the same as others*. *I can afford the same workload as others*. *(laughing)*

(P10)

### 3.4. Nasogastric feeding as a part of life

After facing nasogastric feeding and its effects, the CD patients gradually adapted, making peace with the self-administration of nasogastric feeding and considering nasogastric feeding as a part of their life.

#### 3.4.1. Becoming accustomed to tube insertion

After adopting nasogastric feeding for a period of time, CD patients gradually got used to the physiological changes and the effects of tube feeding and considered nasogastric feeding to not be a big deal.

*After several trials*, *I gradually got used to the tube insertion; it was no big deal… just like a series of mechanical actions*. *One*, *two*, *three*, *insert in…stop when the length reached 55 (cm)*, *and then fix the tube…it was as simple as wearing clothes*.

(P5)

Meanwhile, the CD patients felt normal when they saw that peer patients had tubes and could treat them as a normal occurrence.

*Nowadays*, *nasogastric feeding is more and more common*. *It is no longer strange to see people with a feeding tube in an outpatient department during the day*. *Thus*, *I went to my office with the tube in the daytime as well and found nothing special*. *Actually*, *if you get used to it*, *it would be fine*.

(P8)

Initially, the CD patients felt abnormal with the tube on and only felt normal after they removed the tube. However, after a period of adaption, the feeling of tube insertion gradually became vague, and they felt the same with or without the tube.

*Over time*, *you feel vague about the tube*. *Anyway*, *you do the same thing every day*, *and once you get used to it*, *it becomes nothing*.

(P3)

#### 3.4.2. Taking nasogastric feeding for granted

Initially, the CD patients thought that nasogastric feeding would affect their normal life to a certain extent. After taking actions to adapt to life with the self-administration of nasogastric feeding, a new normal was formed, and they considered nasogastric feeding as simple and convenient as eating.

*I felt it as normal as eating with nothing special and I have gotten used to it now*. *After “having meals”*, *the nasogastric tube should be cleaned*, *just the same as the chopsticks and dishes should be washed after a meal*.

(P7)

After getting used to life with nasogastric feeding, the CD patients did not worry about going out with the tube.

*If someone asks*, *just say that I am sick and need a nutrient solution for feeding*. *And if they look at me*, *just let it be*. *I just think that they are looking at a pretty girl (laughing)…no psychological burden or pressure*.

(P3)

Moreover, the gazes from others seemed to become “normal” as well.

*I would go shopping or go to watch movies with the tube on and I didn’t wear masks later*. *People were relatively calm when seeing me and there were no curious stares to see what’s wrong with me*.

(P11)

## 4. Discussion

In the present study, many CD patients rejected the self-administration of nasogastric feeding therapy due to a fear of tube insertion and concerns about disease severity, frequent hospital visits, dietary restrictions, image, and activity restrictions, which might be associated with a lack of experience and insufficient understanding. These findings were also supported by Chopy et al [27]. A lack of knowledge is associated with the generation of fear [28], and personal direct experience has a significant impact on self-efficacy [29]. Therefore, the self-administration of nasogastric feeding activities must be organized for CD patients to enable them to experience the procedure, which will improve their understanding and reduce fear. Moreover, consistent with findings from other studies [30, 31], the establishment of a group of patients with CD in the present study not only allowed the patients to share their experiences with the self-administration of nasogastric feeding but also created a sense of psychological support. The abnormal feelings regarding nasogastric feeding were significantly reduced, and thus the acceptance of nasogastric feeding was enhanced. Therefore, we propose that peer support should be a part of standard care throughout the entire journey of nasogastric feeding. According to Jukic et al., the theme “acceptance of therapy” must be addressed by intentional training and education before and during discharge from the hospital [32]. Accordingly, health care providers should pay more attention to strategies that promote the acceptance of nasogastric feeding therapy as early as possible.

The need for information was an important issue for CD patients [21]. Because of a lack of understanding, several concerns were generated that resulted in a rejection of self-administration of nasogastric feeding. However, only 8–16% of patients were satisfied with the amount of information they had received [33]. Although our study indicated that the knowledge of self-administration of nasogastric feeding provided by the medical staff was sufficient to meet the needs of CD patients and that they had a high level of trust in health care providers, most patients with CD still had a low awareness of this therapy [34]. Moreover, these patients experienced difficulties in accessing adequate, reliable and practical information [35]. This phenomenon may be associated with the absence of nurses working specifically in advanced roles with CD patients, whereas nurses in the United States and the United Kingdom make significant contributions to the care of these patients [36]. A study conducted in different industrialized and European countries also revealed the importance of patient education for home EN care [37] as these patients struggle to live a normal life. Thus, we highlight the necessity of introducing nurse specialists in China to play this role.

In addition, CD patients who experienced a first successful tube insertion were more confident in self-inserting the tube. Thus, the success rate of the first attempt at tube insertion must be improved. Compared with the conventional nasogastric tube size, a smaller diameter tube could improve the success rate of first-time insertion [38]. In addition, the implementation of measures such as professional technical advice and counseling on the self-administration of nasogastric feeding could improve the success rate.

In our study, the dietary change caused substantial stress to CD patients who were self-administering nasogastric feeding, consistent with the findings reported by Sarlo et al. [31]. During the EN support period, CD patients struggled between their appetite needs and dietary restrictions. This struggle was a particular issue in China, a country with rich culinary cultures. Since food plays an important social role, patients lacked understanding and support regarding their inability to eat normally, which aggravated the psychological burden of CD patients who self-administered nasogastric feeding. This finding is consistent with a previous study showing that a few participants reported that friends or family members had difficulty understanding this abnormal dietary behavior or considered it an eating disorder [21, 39]. According to a survey conducted in Australia, 22% of patients preferred not to eat outside their homes at all [40]. In the study by Liley and Manthorpe, patients stopped eating outside their homes because they did not want anyone to know they were receiving EN support therapy [41]. Combined with our results, CD patients were more withdrawn and less social, or even changed their circle of friends, which in turn resulted in depression or loneliness. These patients have a strong need for respect, understanding, and support [36, 42]. Consequently, families, friends and medical staff should understand them and attempt to create a warm and supportive atmosphere for them. External support was described as a “fantastic” factor that encouraged the CD patients to adjust to lifestyle changes and seek social values, even if they were unable to eat normally [43].

Another important issue was the altered body image caused by the need for nasogastric feeding. In our study, most CD patients who received the nasogastric feeding treatment experienced a substantial change in their self-image, and their self-confidence declined significantly. This finding was consistent with the results of a study conducted by Knowles et al. [44]. Because of the altered body image, CD patients preferred not to go outside and reduced their social activities, which affected their emotional and social functions and quality of life [45]. Currently, relatively few patients are diagnosed with CD in China, and even fewer patients self-administer nasogastric feeding, Therefore, the public does not have good awareness of the disease or nasogastric feeding therapy, which makes the body image of patients with a nasogastric tube vulnerable to stigmatization [46]. Accordingly, the patients’ psychological health may be seriously threatened over time. Furthermore, the nature of the work performed by CD patients might aggravate their psychological pressure, and patients with more severe CD even chose to cut back on their work schedules, change careers, or involuntarily exit the workforce entirely, which in turn again increased their social isolation and had a negative effect on their social relationships [39]. Therefore, medical staff should improve psychological counseling for CD patients who are receiving nasogastric feeding and encourage patients to proactively participate in various social activities to alleviate psychological loneliness. Moreover, the social awareness of nasogastric feeding treatments should be improved to ensure that patients themselves and the general public have a more neutral opinion of the patients’ altered body images.

As shown in a study conducted by Halliday et al., some CD patients thought they benefited greatly from the self-administration of nasogastric feeding and even described it as a “lifeline” [47]. This finding is consistent with the results from our study showing that CD patients thought that nasogastric feeding significantly improved common CD symptoms, such as diarrhea, weight loss and anemia, and reduced the flares of the disease. However, self-administration of the nasogastric feeding treatment was a double-edged sword that also caused many disturbances and inconveniences in their lives. In addition, since the disturbances appeared earlier than the efficacies and these disturbances were long lasting, the patients’ adherence to the self-administration of nasogastric feeding was substantially affected. Furthermore, if the adherence to nasogastric feeding decreased, the efficacies were not guaranteed. For CD patients, a desire to return to normal was the main motivation for them to insist on the therapy, and adherence to nasogastric feeding was improved if they noticed its benefits. Therefore, medical staff should affirm the efficacy of nasogastric feeding as early as possible. Conversely, in light of their current life circumstances and health status, CD patients should redefine “normal” [45]. Some CD patients even explained how they incorporated the self-administration of nasogastric feeding into their daily lives by comparing the procedures to routines such as “brushing my teeth” and “washing up before going to bed” [42]. In our study, some CD patients conveyed a positive attitude and accepted the changes in their bodies and lives. After they adapted to a life including the self-administration of nasogastric feeding and a new normalcy had been rebuilt, the altered image caused by tube insertion was no longer an issue. Winkler et al. also reported the same result that tube acceptance increased with time, and CD patients gradually began to care less about how others looked at them [45]. We believe that a positive attitude toward life is the key to a return to normalcy. Clinicians and nurses should help CD patients come to accept that a period of adaption occurs prior to achieving a better quality of life.

This study has some limitations. First, theme saturation was a relative concept that was limited only to the findings of this study and might change over time. In addition, the participants involved in our study had a relatively high education level, and they were relatively young; thus, they do not represent a general CD population. As indicated in the study, CD patients self-administering nasogastric feeding had a complicated psychological journey and experienced different pressures. Further studies on patient adherence and psychological interventions should be conducted to provide more evidence and a basis for clinical care.

## 5. Conclusions

This study aimed to explore the perceptions and understanding of the experiences of self-administration of nasogastric feeding from the perspective of CD patients. Patients with CD who self-administer nasogastric feeding face many psychological challenges while simultaneously suffering from physical discomfort, an abnormal diet, changes in body image and many disturbances in daily life. Many CD patients eventually adapt to life including the self-administration of nasogastric feeding by proactive self-adjustments; however, not every patient is able to adapt. Therefore, social support offered by health care providers and other patients with CD should be used to help these patients adapt to their “new lives” as soon as possible.
